# Bridging the Knowledge and Training Gap Between Educational Needs and Practices of Orthodontists and General Dental Practitioners Towards Clear Aligner Therapy in India: A Cross-Sectional Survey Study

**DOI:** 10.7759/cureus.100282

**Published:** 2025-12-28

**Authors:** Greeshma Gothankar, Sunil Kumari, Jitender Machawal, Garima Arora, Shailendra Singh Rana

**Affiliations:** 1 Orthodontics, Mahatma Gandhi Mission Dental College and Hospital, Navi Mumbai, IND; 2 Orthodontics, All India Institute of Medical Sciences, Rishikesh, IND; 3 Orthodontics, Central Hospital Kalla, New Delhi, IND; 4 Orthodontics, Manav Rachna Dental College, Faridabad, IND; 5 Orthodontics, All India Institute of Medical Sciences, Bathinda, IND

**Keywords:** clear aligner therapy, dental education, knowledge-attitude-practice, orthodontics, patient compliance

## Abstract

Background: Clear aligner therapy (CAT) has rapidly evolved as a preferred orthodontic treatment option due to its aesthetic appeal, comfort, and removability. Despite increasing availability, variability exists in practitioners' knowledge, attitudes, and practical application of CAT.

Objective: This study aimed to determine the knowledge and demand for CAT education amongst practitioners so as to offer insights for future tailored curricula on CAT.

Methods: A cross-sectional survey was conducted among 112 licensed orthodontists and general dentists. Data were collected using a validated, self-administered questionnaire that focused on CAT-related knowledge, attitudes, practices, and training needs. Descriptive and inferential statistics were analyzed using SPSS Version 26 (IBM Corp., Armonk, NY, USA), with significance set at p < 0.05.

Results: Participants had a mean age of 33.97 years, with 73.2% (n = 89) being orthodontists. While 60.7% (n=68) had received CAT training, and 57.1% (n=64) had access to CAT systems within their clinics, 41.1% (n=41) reported no clinical experience with CAT. Major barriers identified included cost (77.7%, n = 87) and patient compliance (46.4%, n = 52). Most respondents recognized CAT as suitable for minor to moderate malocclusions and polyurethane as the standard material used in this context. A majority acknowledged the need for fewer clinical visits with CAT compared to traditional braces. Practitioners expressed a willingness to pursue further CAT training and integrate it into their routine practice. Statistical analyses revealed significant associations between access to CAT systems and confidence in patient education and clinical application.

Conclusion: This study highlights the gaps between theoretical knowledge and the clinical application of CAT, with cost and patient compliance being key challenges that must be addressed. The findings underscore the importance of targeted continuing education in enhancing the adoption of CAT and optimizing patient care in modern orthodontics.

## Introduction

Clear aligner therapy (CAT) is a removable and aesthetic alternative to conventional fixed appliances. It can treat a wider range of malocclusions due to continuous improvements in aligner materials, digital workflows, and three-dimensional treatment planning [1,2]. Despite this, acceptance of CAT varies and depends on practitioners’ knowledge, clinical training, and confidence in aligner systems. Although the brands such as Invisalign, Spark and Clear-Correct are easily available in the market, their successful utilization requires thorough knowledge of case selection, biomechanics, and patient management [3]. In India, undergraduate and postgraduate orthodontic curricula continue to focus on traditional fixed appliance therapy, with limited importance to aligner-based treatment modalities [4]. This educational limitation may contribute to inconsistent levels of knowledge and variable clinical utilization of CAT among practitioners. Some practitioners use aligners, whereas others still follow specifically conventional fixed braces due to the high cost of aligners, patient compliance issues, or a lack of experience [5]. Assessing these differences through a structured knowledge, attitude, and practice (KAP) framework provides valuable insights into practitioners’ preparedness and the barriers to broader adoption [6].

Although international studies have explored dental professionals’ perceptions of aligner therapy, there is limited evidence from India addressing these parameters. Therefore, this study aimed to determine the knowledge and demand for CAT education among orthodontists and general dental practitioners in India so as to offer insights for future tailored curricula on CAT. Identifying existing gaps will also help inform targeted continuing dental education programs and facilitate evidence-based integration of aligner therapy into modern orthodontic practice [7].

## Materials and methods

Study design and participants

This study employed a cross-sectional, descriptive survey design to evaluate the knowledge, attitudes, practices, and educational demands of orthodontic practitioners regarding Clear Aligner Therapy (CAT). The survey was carried out using an online, closed-ended questionnaire, administered via Google Form for broad geographic coverage. The target population comprised licensed orthodontic practitioners and general dentists involved in orthodontic care. Ethical approval was obtained from the Institutional Review Board before study commencement. The ethical approval number was (MRIIRS/MRDC/SDS/IEC/2025/114). Informed consent was obtained from all participants, and confidentiality of responses was strictly maintained throughout the study.

Inclusion and exclusion criteria

Licensed orthodontists and postgraduate students or general dentists involved in orthodontic treatment who were willing to participate voluntarily and gave informed consent were included in the study. Undergraduate or interns and Incomplete survey responses were excluded from the study.

Sample size determination

The sample size was calculated using the formula for estimating proportions with a 95% confidence level, assuming a prevalence of 50% to maximize the sample size and a margin of error of 10%. This calculation yielded a minimum required sample size of 97 participants. To account for a potential 10% non-response rate, the sample size was increased to 107 participants to ensure adequate statistical power and representativeness. A convenience sampling method was employed, inviting orthodontic practitioners from various clinical settings, including private practices, government institutions, and academic centers, to participate in the study.

Survey instrument

A structured and validated questionnaire was developed for online distribution. It was divided into five sections:

Section A: Demographic and professional details (e.g., age, gender, years of experience, specialty, and practice setting).

Section B: Knowledge-based questions related to CAT, such as clinical indications, materials used, biomechanics, and treatment protocols.

Section C: Assessment of attitudes towards the adoption and effectiveness of CAT, using a five-point Likert scale.

Section D: Practice-oriented queries, including frequency of aligner usage, system preference, patient management, and challenges encountered.

Section E: Evaluation of learning needs related to CAT, including treatment planning, case selection, and interdisciplinary approaches.

The questionnaire was reviewed for content validity by three subject experts in orthodontics. A pilot test involving 10 respondents was conducted to assess clarity, relevance, and reliability, following which necessary modifications were made. The Item-level Content Validity Index (I-CVI) values ranged from 0.80 to 1.00, and the Scale-level CVI (S-CVI/Ave) was 0.93, indicating excellent content validity of the instrument.

Data collection and analysis

The finalized questionnaire was converted into an online format using Google Forms and disseminated through professional dental networks, email groups, and social media platforms like WhatsApp and LinkedIn. The survey link was accompanied by an introductory statement explaining the study’s purpose, voluntary participation, and confidentiality assurance. Participants provided informed consent before accessing the questionnaire. Measures were taken to prevent multiple submissions and maintain the anonymity of respondents.

Data were automatically recorded in Google Sheets and then exported to Microsoft Excel for cleaning and coding, and the analysis was conducted using SPSS Version 26 (IBM Corp., Armonk, NY, USA). The descriptive analysis was done, and the data were presented in terms of frequency and percentage. Wherever required, the mean and median were calculated. The comparison was done using the Chi-Square test, and a P-value of less than 0.05 was considered significant.

## Results

**Figure 1:**
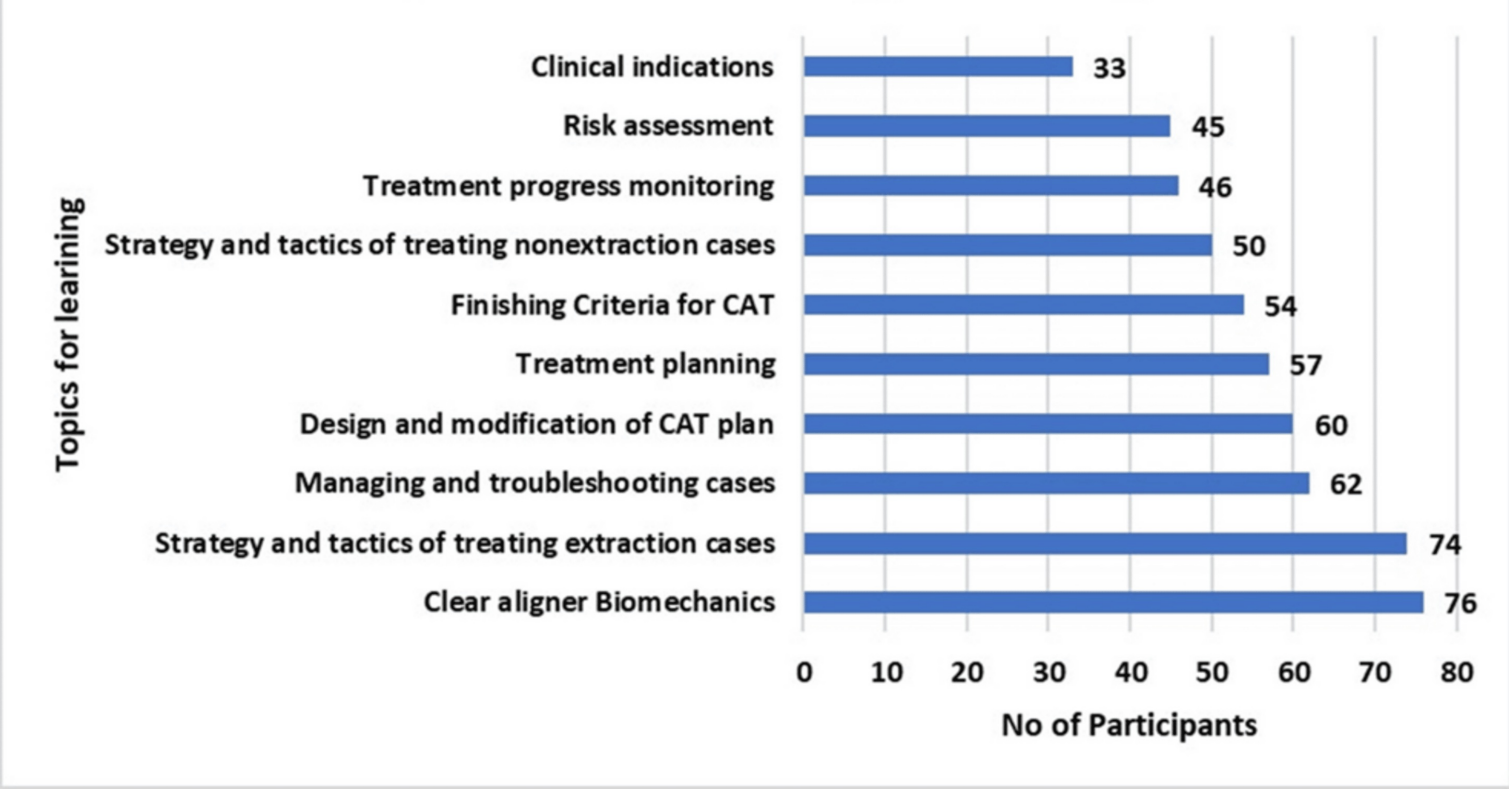
Topics for further learning and training

The questionnaire was distributed to 125 practitioners via social media and email, of whom 112 participants responded. A total of 112 practitioners participated in the study. The demographic characteristics of the respondents are summarized in Table 1. Experience with clear aligner therapy, including training status, access to aligner systems, and challenges encountered in clinical use, is presented in Table 2. Responses related to practitioners’ knowledge, attitudes, and practices toward clear aligner therapy are detailed in Table 3. Practitioners’ understanding of the magnitude of tooth movements achievable using clear aligners is summarized in Table 4. Areas identified by participants for further learning and training are illustrated in Figure 1. All the participants (100%) agreed that patient compliance is essential for success in CAT therapy.

**Table 1:** General characteristics of the participants. CAT: Clear aligner therapy

Variable	Frequency (n = 112)	Percentage (%)
Gender		
Female	50	44.64
Male	62	55.36
Age		
< 30	37	33.04
30 to 35	39	34.82
> 35	36	32.14
Occupation		
Orthodontist	89	79.46
General Dentist	23	20.54
Working Sector		
Private	79	70.54
Government	23	20.54
Semi-Government	5	4.46
PG Student	5	4.46
Experience (Years)		
0 to 1	20	17.86
2 to 5	51	45.54
> 5	41	36.61

**Table 2:** Experience using CAT CAT: Clear aligner therapy

Variable	Frequency (n = 112)	Percentage (%)
Experience using CAT (in years)		
0	46	41.07
1 to 2	38	33.93
2 to 4	18	16.07
> 4	10	8.93
Training in CAT	68	60.71
Access to a clear CAT in the Clinic	64	57.14
The CAT System used in the clinic.		
Other	70	62.50
Invisalign	50	44.64
Clear Correct	10	8.93
Spark	8	7.14
Direct Printed CAT	4	3.57
Challenges faced in using CAT		
Cost	87	77.7
Compliance	52	46.4
Training	42	37.5
Case Selection	35	31.3

**Table 3:** KAP related to CAT CAT: Clear aligner therapy *P value calculated using the Chi-square Test; df=Degree of Freedom; Cramér’s V Effect Size: 0.1–0.2: Small; 0.2–0.3: Medium; >0.3: Large

Questions	Variables	Access to clear aligner systems		P Value	Chi-Square	Cramér’s V Effect Size
		Yes (n=64)	No (n=48)			
CAT is best suited for	Minor/Moderate Malocclusion	63	45	0.186	4.813 (df=3)	0.185
	None of the above	0	2			
	Severe skeletal malocclusion	1	0			
	TMD cases	0	1			
Material is commonly used in CAT	None of the above	4	2	0.860	2.574 (df=3)	0.152
	PMMA	11	8			
	Polycarbonate	6	4			
	Polyurethane	43	34			
Treatment with CAT requires fewer visits	No	5	9	0.083	3.000 (df=1)	0.163
	Yes	59	39			
NOT a clear CAT brand	Damon	43	35	0.621	1.771 (df=3)	0.125
	Demon	18	12			
	Invisalign	1	1			
	Spark	2	0			
Attachments or auxiliaries used with CAT	No	2	1	0.399	1.838 (df=2)	0.128
	Not Sure	1	3			
	Yes	61	44			
I believe CAT are as effective as braces	Strongly Agree	17	5	0.251	5.377 (df=4)	0.155
	Agree	24	19			
	Neutral	13	12			
	Disagree	9	10			
	Strongly Disagree	1	2			
CAT is important for modern dental practice	Strongly Agree	29	17	0.096	4.686 (df=2)	0.145
	Agree	32	23			
	Neutral	3	8			
I feel confident explaining CAT to patients	No	4	11	0.000	15.763 (df=2)	0.266
	Sometimes	9	16			
	Yes	51	21			
I am open to integrating CAT into my routine practice	Maybe	5	8	0.340	2.159 (df=2)	0.139
	No	2	1			
	Yes	57	39			
Cost is a major barrier to adopting CAT	Strongly agree	23	27	0.177	4.927 (df=3)	0.149
	Agree	32	17			
	Neutral	6	2			
	Disagree	3	2			
I prefer fixed orthodontic appliances over CAT	Depends on the case	43	22	0.034	6.777 (df=2)	0.195
	No	3	1			
	Yes	18	25			
I would attend training if offered locally or online	Maybe	8	4	0.523	1.296 (df=2)	0.108
	No	1	0			
	Yes	55	44			
Learn treatment planning and biomechanics in CAT	Maybe	3	3	0.914	0.179 (df=2)	0.040
	No	1	1			
	Yes	60	44			
CAT cases treated per month	0	10	36	0.000	42.193 (df=3)	0.434
	1-2	35	11			
	2-4	10	0			
	>4	9	1			
Digital impressions for CAT cases	No	5	19	0.000	26.675 (df=2)	0.388
	Outsourced	5	11			
	Yes	54	18			
Follow up every 4–6 weeks during CAT	No	6	18	0.000	15.225 (df=2)	0.262
	Occasionally	9	9			
	Yes	49	21			
Educate patients about CAT options	Always	47	18	0.000	16.257 (df=2)	0.269
	Never	0	3			
	Sometime	17	27			
Offer clear CAT therapy	No	5	30	0.000	38.182 (df=1)	0.583

**Table 4:** Knowledge of the usage of CAT CAT: Clear aligner therapy

Amount is possible only with CAT	0 mm	1 to 2 mm	2 to 4 mm	> 4 mm	Do not know
Intrusion	1 (0.9)	76 (67.9)	31 (27.7)	2 (1.8)	9 (8.0)
Extrusion	14 (12.5)	70 (62.5)	19 (17.0)	3 (2.7)	12 (10.7)
Molar distalization	6 (5.4)	48 (42.9)	54 (48.2)	5 (4.5)	10 (8.9)
Molar mesialization	17 (15.2)	62 (55.4)	23 (20.5)	4 (3.6)	13 (11.6)
Arch expansion	2 (1.8)	44 (39.3)	47 (41.9)	9 (8.1)	10 (8.9)

## Discussion

This study offers a broad examination of orthodontists ' and dental practitioners' current knowledge, attitudes, behaviors, and educational needs, highlighting key gaps between awareness and clinical application. Although various unambiguous aligner systems are readily accessible, our data show that a substantial number of practitioners (41.1%; n = 46) acknowledged having no previous clinical experience with CAT, despite over 60% having attended training. This discrepancy underscores a disconnection between theoretical understanding and hands-on proficiency, an issue repeatedly noted in global literature [8,9].

In 75% respondents were orthodontists, and others were general practitioners. Given the expanding accessibility of aligner systems and company-led training programs, CAT is increasingly being practiced not only by orthodontists but also by general dental practitioners. In India, where many patients first consult general dentists for orthodontic concerns, their awareness and clinical readiness directly influence treatment uptake and outcomes. Therefore, including both orthodontists and dental practitioners provides a comprehensive assessment of current knowledge, attitudes, and practices related to aligner therapy across diverse clinical settings [9]. Two principal barriers, cost and patient compliance, emerged as the dominant constraints affecting aligner adoption. Nearly three-quarters of the participants cited cost as the main reason for the delayed adoption of aligners in both public and private clinical settings. Likewise, patient compliance remains pivotal given the removable nature of aligners, which, while enhancing comfort and hygiene, places treatment success heavily on patient adherence. These findings are similar to earlier studies emphasizing the behavioral and economic determinants of CAT uptake [10-12]. Clinically, most participants recognized CAT as best suited for mild to moderate malocclusions; these findings corroborate a systematic review that found aligners are less effective in complex cases requiring precise root control or extrusion [13]. From an educational perspective, the study reveals an urgent need for structured, skill-oriented training programs focusing on topics like appropriate case selection and treatment planning, clear aligner biomechanics and tooth movement predictability, digital workflow integration, and managing relapse and troubleshooting refinements. Most participants expressed a strong desire for additional CAT-related training [14-16].

An important dimension of CAT adoption in India is the growing influence of commercial aligner companies, particularly systems such as Invisalign and Clear-Correct, as well as emerging local brands. Aggressive marketing strategies and simplified certification programs have expanded general practitioners' access to aligners. Although it is easily available, it also has some drawbacks, namely uneven quality control, overreliance on specific software solutions, and limited application of sound biomechanical principles. Academic institutions and professional bodies must therefore ensure that clinical decision-making remains evidence-based and independent of commercial bias [14,15]. Introducing clear aligner therapy into the undergraduate and postgraduate dental curricula in India would be beneficial. Dedicated modules covering digital orthodontics, 3D printing, and aligner biomechanics would prepare future clinicians for the evolving landscape of orthodontic care [16].

Limitations

This study's cross-sectional design is limited to causal inference. The reliance on self-reported data may introduce recall and social desirability biases. The findings may not be generalizable due to the restricted geographic area. Additionally, the study did not evaluate treatment outcomes or objectively measure clinical proficiency. Future longitudinal studies with larger samples and objective performance metrics are recommended to validate and expand upon these findings.

## Conclusions

The present study highlights a progressive yet uneven adoption of Clear Aligner Therapy among dental professionals in India. Although dental practitioners have theoretical knowledge, cost barriers, patient compliance issues, and limited hands-on training continue to hinder broader clinical integration. Structured, evidence-based educational initiatives focusing on case selection, biomechanics, and digital workflow are essential for improving competency. Incorporating clear aligner modules into dental curricula and standardized CDE programs will be key to fostering competent, ethically guided adoption of CAT in modern orthodontic practice.
